# Long–term trends in ceftolozane–tazobactam susceptibility among gram–negative pathogens in the United States: a nine–year SMART analysis (2016–2024)

**DOI:** 10.1093/jacamr/dlag134

**Published:** 2026-07-07

**Authors:** Mark G Wise, Karri A Bauer, John S Esterly, Fakhar Siddiqui, Katherine Young, Mary R Motyl, Daniel F Sahm

**Affiliations:** IHMA, 2122 Palmer Drive, Schaumburg, IL 60173, USA; Merck & Co., Inc., Rahway, NJ, USA; Merck & Co., Inc., Rahway, NJ, USA; Merck & Co., Inc., Rahway, NJ, USA; Merck & Co., Inc., Rahway, NJ, USA; Merck & Co., Inc., Rahway, NJ, USA; IHMA, 2122 Palmer Drive, Schaumburg, IL 60173, USA

## Abstract

**Objectives:**

To evaluate trends in the susceptibility of clinical Enterobacterales and *Pseudomonas aeruginosa* from the USA to ceftolozane/tazobactam.

**Methods:**

The SMART surveillance programme collected 30 658 Enterobacterales and 7454 *P. aeruginosa* isolates from 37 unique clinical sites in the USA from 2016 to 2024. Nine sites contributed isolates each year. MICs were determined by broth microdilution testing and interpreted using 2025 CLSI breakpoints. Ceftolozane/tazobactam nonsusceptible Enterobacterales were examined for β-lactamase carriage.

**Results:**

Overall, 93.9% and 95.8% of clinical isolates of Enterobacterales and *P. aeruginosa* were susceptible to ceftolozane/tazobactam, respectively. Considering all participating sites, annual percent susceptible values for ceftolozane/tazobactam against Enterobacterales ranged from 95.3% (2018, *n* = 3657) to 92.0% (2024, *n* = 3249) with a statistically significant decreasing trend (*P* < 0.001; Cochran-Armitage test). Limiting analysis solely to the nine clinical sites that participated in each year confirmed this trend [range: 94.5% susceptible (2019, *n* = 1609) to 91.4% susceptible (2024, *n* = 1544); *P* = 0.007]. Including all sites, *P. aeruginosa* percent susceptible values for ceftolozane/tazobactam remained consistent from 2016 (95.0%) to 2024 (95.0%) [range: 94.5% (2017, *n* = 991) to 96.9% (2020, *n* = 817)]. However, considering solely the consistently contributing sites, the rate of ceftolozane/tazobactam susceptibility increased [*P* = 0.013; range: 91.9% (2017, *n* = 371) to 96.4% (2024, *n* = 391)]. The incidence of NDM metallo-β-lactamase and CTX-M ESBLs among ceftolozane/tazobactam nonsusceptible Enterobacterales each exhibited a statistically significant increasing trend.

**Conclusions:**

From 2016 to 2024, ceftolozane/tazobactam activity against US *P. aeruginosa* remained consistently high; however, a trend of marginally decreasing susceptibility to ceftolozane/tazobactam among Enterobacterales was observed, likely associated with increased incidence of NDM- and CTX-M-producers.

## Introduction

Antimicrobial resistance among Enterobacterales and *Pseudomonas aeruginosa* continues to erode the reliability of standard empiric and targeted regimens in US hospitals. Resistant Enterobacterales, including ESBL-, AmpC- and carbapenemase-producers, and intrinsically resistant *P. aeruginosa* together account for a large share of complicated urinary tract, intra-abdominal, respiratory and bloodstream infections.^1^ Guidance documents emphasize the clinical and stewardship importance of distinguishing phenotypes such as ESBL-producing Enterobacterales and difficult-to-treat resistant (DTR) *P. aeruginosa*, because these categories meaningfully narrow effective β-lactam options and are associated with worse patient outcomes.^1^

Ceftolozane/tazobactam (C/T) was developed to address these challenges by pairing the anti-pseudomonal cephalosporin, ceftolozane, with a traditional β-lactamase inhibitor, tazobactam, to restore activity against many Ambler class A β-lactamase–producing Enterobacterales while retaining potent antibacterial activity against *P. aeruginosa*. C/T was initially approved in the USA for complicated urinary tract and complicated intra-abdominal infections, followed by an expanded indication for hospital-acquired bacterial pneumonia and ventilator-associated bacterial pneumonia.^2^ As clinical use increased, particularly against multidrug-resistant (MDR) *P. aeruginosa*, the need for robust, longitudinal susceptibility surveillance became more acute.

Large surveillance networks have consistently demonstrated high *in vitro* activity of C/T against US *P. aeruginosa* isolates, including subsets with resistance to standard-of-care β-lactams. For example, pre-approval, the PACTS programme across 32 US hospitals in 2012–2015 reported excellent C/T activity against broad collections of *P. aeruginosa*, including isolates from respiratory infections in intensive care units and organisms meeting MDR or DTR-like criteria.^3^ In contrast, C/T susceptibility patterns among Enterobacterales are more heterogeneous by species and resistance mechanisms, likely driven by the shifting prevalence of ESBL-, AmpC- and carbapenemase-producing subpopulations.^4^

The SMART (Study for Monitoring Antimicrobial Resistance Trends) global surveillance programme is an ongoing international initiative that monitors trends in antimicrobial resistance among clinically important Gram-negative bacterial pathogens.^5^ The purpose of this study was to perform longitudinal analyses (i.e. investigate annual trends) of ceftolozane/tazobactam susceptibility among clinical isolates of Enterobacterales and *P. aeruginosa* collected in the USA by SMART from 2016 to 2024, including molecular analysis of the Enterobacterales nonsusceptible to C/T, and to contextualize these findings relative to comparator antipseudomonal and anti-Enterobacterales agents used in contemporary practice.

## Methods

### Bacterial isolates

Each US clinical laboratory site that participated in the SMART programme was asked to collect consecutive, clinically significant isolates of aerobic or facultatively anaerobic Gram-negative bacilli from intra-abdominal infections (IAI), respiratory tract infections (RTI), urinary tract infections (UTI) and, starting in 2018, bloodstream infections (BSI). Isolates were restricted to one isolate per patient per Gram-negative species per year. All isolates were transported to a central laboratory (IHMA, Schaumburg, IL, USA), where they were re-identified using matrix-assisted laser desorption ionization-time of flight (MALDI-TOF) mass spectrometry (Bruker Daltonics, Billerica, MA, USA) prior to antimicrobial susceptibility testing.

From 2016 to 2024, the SMART surveillance programme collected a total of 30 658 evaluable Enterobacterales and 7454 *P. aeruginosa* isolates from 37 unique clinical sites in 25 US states; however, not all sites participated each year. Table S1 (available as Supplementary data at *JAC-AMR* Online) provides the number of Enterobacterales and *P. aeruginosa* isolates collected over this time period by state and clinical site. In total, nine clinical sites in seven states (CA [two sites], CO, IL, NY [two sites], OH, WA and WI) were consistent annual contributors to the programme each year from 2016 to 2024.

### Antimicrobial susceptibility testing

The Clinical and Laboratory Standards Institute (CLSI) reference broth microdilution method was used to determine isolate MICs.^6^ MICs were interpreted as susceptible, intermediate or resistant using 2025 CLSI M100 breakpoints.^7^ Isolates were classified as MDR based on resistance to ≥3 sentinel agents in differing drug classes (amikacin [aminoglycosides], aztreonam [monobactams], cefepime [4th-generation cephalosporin], ceftazidime [3rd-generation cephalosporin; considered for Enterobacterales only], colistin [polymyxins], meropenem [carbapenems], levofloxacin [fluoroquinolones] and piperacillin/tazobactam [penicillin/β-lactamase inhibitor combinations]).^8^ Difficult-to-treat resistance (DTR) phenotypes for *P. aeruginosa* were categorized using the criteria published by Kadri *et al.*^9^ Specifically, DTR phenotypes were defined by isolates not susceptible (intermediate or resistant) to all tested β-lactams (aztreonam, ceftazidime, cefepime, imipenem, meropenem, piperacillin-tazobactam), as well as fluoroquinolones (levofloxacin). The DTR definition excluded newer β-lactam/β-lactamase inhibitor combinations like C/T.

### Statistical analysis

The Cochran-Armitage test was used to assess linear trends in percentage susceptible values from 2016 to 2024 using XLSTAT v2024.2.2. A two-tailed *P*-value <0.05 was considered statistically significant.

In order to investigate whether susceptibility trends changed direction or rate over the 9-year period, the Joinpoint regression programme (v6.0.1)^10^ was employed utilizing a maximum of one joinpoint to avoid overfitting.

### Screening for β-lactamase genes

Enterobacterales nonsusceptible to C/T (MIC ≥4 mg/L) were screened for acquired β-lactamase genes; for isolates collected in 2016–2022, published multiplex PCR assays were used to screen for β-lactamase genes as described previously.^11,12^ For those collected in 2023, isolates were characterized by short-read whole-genome sequencing (Illumina HiSeq 2 × 150 bp reads) to a targeted coverage depth of 100×^13^ and analyzed using the CLC Genomics Workbench (Qiagen), and the Resfinder database was used to detect β-lactamase genes.^14^ For those collected in 2024, molecular characterization was carried out using whole genome sequencing by PromethION 2 Integrated (Oxford Nanopore Technologies, Oxford, UK) using the R10.4.1 flow cell. Base calling was performed using the super accurate model (dorado 7.6.7). Assembly was conducted using the EPI2ME isolates workflow version 1.4.1. Antimicrobial resistance markers were identified using AbritAMR.^15^ The deduced amino acid sequences of identified β-lactamase genes were extracted so that variants could be determined using the NCBI Bacterial Antimicrobial Resistance Reference Gene Database (Bioproject 313047). Due to budget restrictions, in some years not all C/T nonsusceptible isolates were molecularly characterized.

## Results

From 2016 to 2024, clinical laboratories in the US participating in the SMART global surveillance programme collected a total of 30 658 evaluable Enterobacterales and 7454 *P. aeruginosa*. Overall, 93.9% of the Enterobacterales were susceptible to C/T at the CLSI 2025 breakpoint (Table 1). Meropenem also inhibited >90% of the isolates. C/T inhibited 98.2% and 94.4% of the *Escherichia coli* and *Klebsiella pneumoniae* collected, in each case a higher percentage than comparator cephalosporins. C/T displayed reduced activity versus taxa expected to carry intrinsic AmpC-type enzymes, including the *Enterobacter cloacae* complex (74.1% S) and *Citrobacter freundii* complex (77.8% S). However, against the *Morganellaceae*, C/T inhibited 97.9% of the isolates, the second most active agent after meropenem (99.6% S).

**Table 1.** Percent susceptible values for ceftolozane/tazobactam and comparator agents against clinical isolates of Enterobacterales and *P. aeruginosa* collected in the USA from 2016 to 2024

		% Susceptible^a^							
Group	*n*	C/T	IPM	MEM	CAZ	FEP	TZP	CRO	LVX
All Enterobacterales	30 658	93.9	89.9	98.8	84.3	88.1	87.1	79.6	60.5
*Escherichia coli*	12 305	98.2	99.4	99.6	85.5	85.3	92.2	80.8	53.5
*Klebsiella pneumoniae*	5654	94.4	96.5	97.3	82.9	83.9	83.3	80.8	61.1
*Enterobacter cloacae* complex ^b^	2354	74.1	92.2	97.9	66.9	85.0	69.2	61.1	65.5
*Citrobacter freundii* complex ^c^	862	77.8	92.8	97.6	70.3	93.0	72.3	64.8	61.9
*Morganellaceae* ^d^	2817	97.9	34.1	99.6	92.3	94.7	97.4	88.2	54.1
MDR	3204	55.4	83.0	89.5	5.0	24.9	35.5	2.7	21.3
All *P. aeruginosa*	7454	95.8	68.4	78.1	78.8	79.6	74.9	na	66.8
CAZ-nonsusceptible	1581	81.1	41.5	47.9	0.0	20.6	8.7	na	44.1
FEP-nonsusceptible	1520	81.0	37.2	41.6	17.4	0.0	12.8	na	36.0
TZP-nonsusceptible	1872	85.1	42.5	46.6	22.9	29.2	0.0	na	40.1
MEM-nonsusceptible	1630	85.8	8.2	0.0	49.4	45.6	38.7	na	29.0
MDR	1116	75.5	22.7	23.9	14.2	9.7	5.5	na	23.9
DTR	527	68.7	0.0	0.0	0.0	0.0	0.0	na	0.0

^a^Susceptiblity by CLSI 2025 criteria.

^b^Includes (*n): Enterobacter asburiae (148), Enterobacter bugandensis (158), Enterobacter cancerogenus (1), Enterobacter cloacae (1296), Enterobacter cloacae complex (61), Enterobacter hormaechei (438), Enterobacter kobei (114), Enterobacter ludwigii (59), Enterobacter roggenkampii (43), Enterobacter xiangfangensis (36).*

^c^Includes (*n*); *Citrobacter braakii (72), Citrobacter freundii (767), Citrobacter gillenii (1), Citrobacter sedlakii (21), Citrobacter youngae (1).*

^d^Includes (*n*): *Morganella morganii (478), Proteus hauseri (57), Proteus mirabilis (1811), Proteus penneri (16), Proteus sp (47), Proteus vulgaris (81), Providencia alcalifaciens (2), Providencia rettgeri (129), Providencia sp (6), Providencia stuartii (190).*

C/T, ceftolozane/tazobactam; IPM, imipenem; MEM, meropenem; CAZ, ceftazidime; FEP, cefepime; TZP, piperacillin/tazobactam; CRO, ceftriaxone; LVX, levofloxacin; MDR, multidrug resistant; DTR, difficult-to-treat resistant; na, not applicable.

Against *P. aeruginosa*, 95.8% of the US isolates were susceptible, approximately 16–17 percentage points higher than for the antipseudomonal cephalosporins (ceftazidime and cefepime) and meropenem (Table 1). Overall, 25.1%, 21.2% and 20.4% of the *P. aeruginosa* collected tested nonsusceptible to piperacillin/tazobactam, ceftazidime and cefepime, respectively. Ceftolozane/tazobactam inhibited 85.1% of piperacillin/tazobactam-nonsusceptible, 81.1% of ceftazidime-nonsusceptible and 81.0% of cefepime-nonsusceptible isolates. Overall, 21.9% of isolates were nonsusceptible to meropenem, and 85.8% of those were susceptible to ceftolozane/tazobactam. In total, 1116 (15.0%) and 527 (7.1%) *P. aeruginosa* isolates were identified as MDR and DTR, respectively. Against these more resistant subsets, ceftolozane/tazobactam inhibited 75.5% of MDR and 68.7% of the DTR phenotypes.

**Figure 1.**
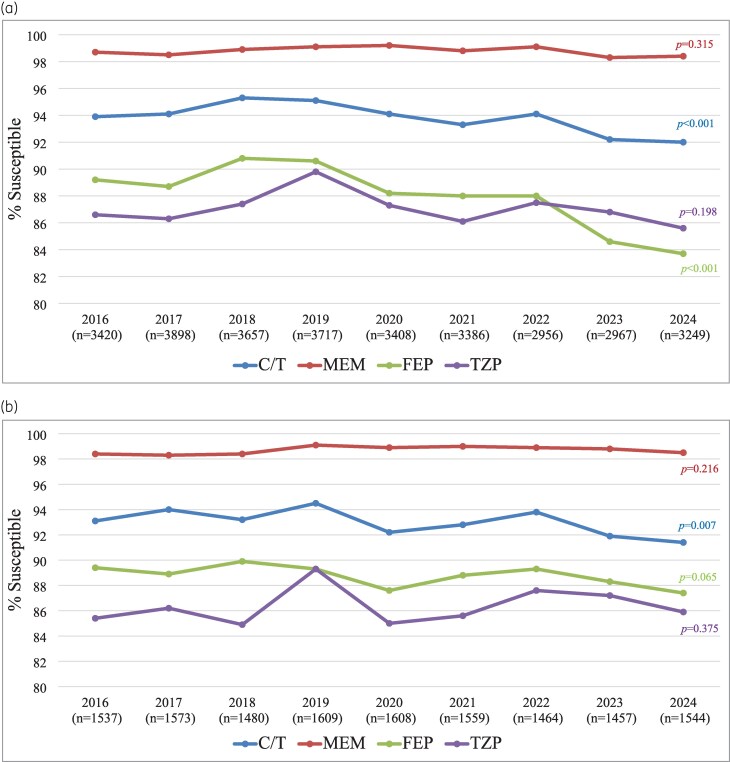
Longitudinal trends from 2016 to 2024 in the percentage of Enterobacterales isolates testing as susceptible to the indicated agents among (a) all isolates collected in the USA for the SMART programme and (b) isolates collected solely from the clinical sites that participated in the programme each year from 2016 to 2024. Significance in trend over time was determined by the Cochran–Armitage test: two-tailed *P*-value shown in the figure (*P* < 0.05 was considered statistically significant). Percentages presented in this figure are provided in Table S2. Abbreviations. C/T, ceftolozane/tazobactam; MEM, meropenem; FEP, cefepime; TZP, piperacillin/tazobactam.

Trends in Enterobacterales annual ceftolozane/tazobactam-susceptible percentages, as well as those of comparator agents (meropenem, cefepime and piperacillin/tazobactam) over the 9-year period from 2016 to 2024 are shown in Figure 1. As there was considerable variability regarding the hospitals that participated in the SMART programme over this time period leading to potential bias (see Table S1 for site participation), the longitudinal analysis was performed twice, once including the totality of isolates collected, and a second time considering solely the isolates from the clinical sites that contributed each year. Evaluating all isolates (Figure 1a), the percent susceptible value for C/T ranged from 95.3% in 2018 to 92.0% in 2024, demonstrating a statistically significant decrease in susceptibility over time (*P* < 0.001; Cochran–Armitage test for trend). Using the Joinpoint piecewise regression model, the susceptibility decrease appeared to begin in 2018 and continued to 2024 (Figure S1A). Susceptibility to cefepime also decreased over time (*P* < 0.001), while no significant trends were observed for meropenem or piperacillin/tazobactam (*P* *>* 0.05 for both). Evaluating isolates solely from the consistently contributing sites yielded similar results (Figure 1b); the percent susceptible value to C/T ranged from 94.5% in 2019 to 91.4% in 2024, with a statistically significant linear decreasing trend (*P* = 0.007). In this case, Joinpoint regression suggested that the rate of decrease in susceptibility over the nine-year time frame was constant, as no change point was detected (Figure S1B). No significant linear trends were observed among comparators.

**Figure 2.**
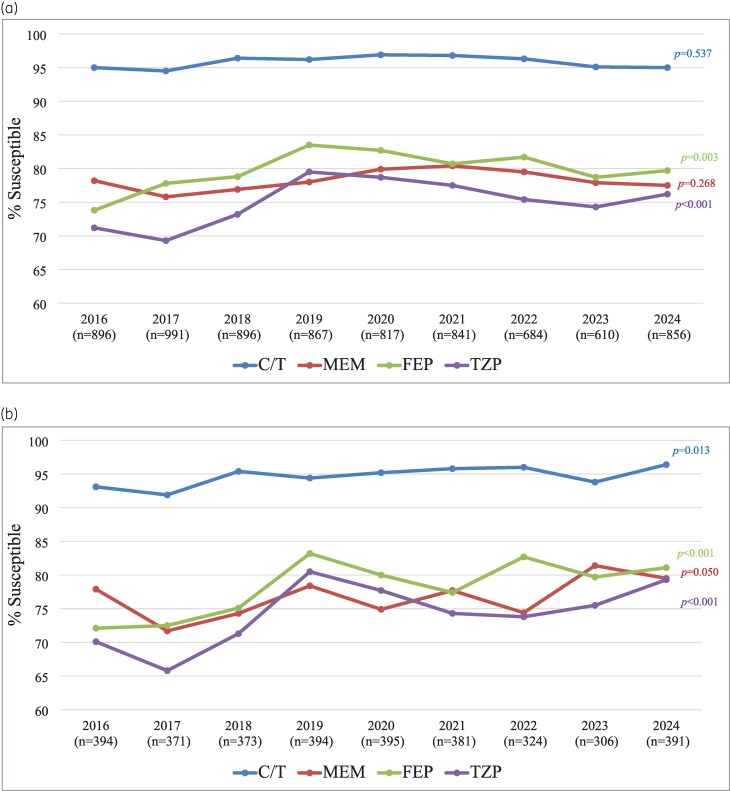
Longitudinal trends from 2016 to 2024 in the percentage of *P. aeruginosa* isolates testing as susceptible to the indicated agents among (a) all isolates collected in the USA for the SMART programme and (b) isolates collected solely from the clinical sites that participated in the programme each year from 2016 to 2024. Significance in trend over time was determined by the Cochran–Armitage test: two-tailed *P*-value shown in the figure (*P* < 0.05 was considered statistically significant). Percentages presented in this figure are provided in Table S3. Abbreviations. C/T, ceftolozane/tazobactam; MEM, meropenem; FEP, cefepime; TZP, piperacillin/tazobactam.

In contrast, annual trends in C/T susceptibility among the *P. aeruginosa* collected in the USA, including all sites (Figure 2a) and only the consistently contributing sites (Figure 2b), did not reveal evidence for increasing resistance. Considering all sites, susceptibility to C/T ranged from 94.5% (2017) to 96.9% (2020) with no significant trend (*P* = 0.537; Cochran–Armitage test for trend). Joinpoint analysis of this data set, however, suggested that C/T susceptibility increased slightly from 2016 to 2020, followed by a slight decrease (Figure S2A). Among comparators, a linear trend of increasing susceptibility to both cefepime (*P* = 0.003) and piperacillin/tazobactam (*P* < 0.001) was noted. When only the *P. aeruginosa* isolates from the consistently contributing sites were examined, trends of increasing susceptibility over time to all four agents were observed. Joinpoint analysis of C/T susceptibility did not identify a significant change point in this data set (Figure S2B).

**Figure 3.**
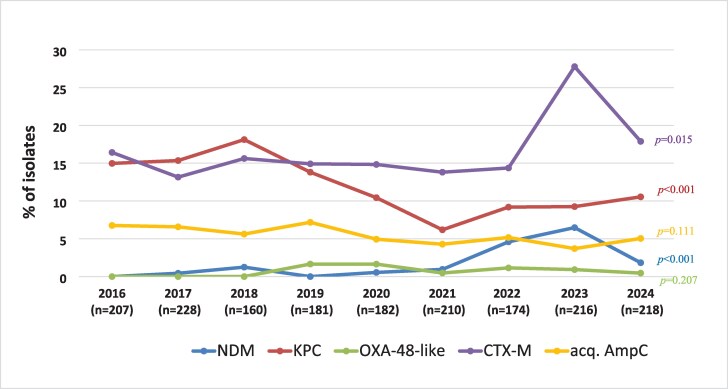
Acquired β-lactamase detection rate* among C/T-nonsusceptible Enterobacterales in the US, 2016–2024. Significance in trend over time was determined by the Cochran–Armitage test: two-tailed *P*-value shown in the figure (*P* < 0.05 was considered statistically significant). Percentages presented in this figure are provided in Table S4. * β-lactamases expected to be chromosomal (e.g. AmpCs in *Enterobacter* spp., etc.) not shown.

In an effort to understand the underlying molecular mechanisms that may be responsible for the decreasing susceptibility to C/T among the Enterobacterales in the USA, C/T nonsusceptible isolates were interrogated for their acquired β-lactamase carriage each year (Figure 3). Considering all sites that participated, the rate of CTX-M detection was relatively stable from 2016 (16.4%) to 2022 (14.4%), followed by a sharp spike in 2023 (27.8%) and a subsequent reversion in 2024 (17.9%), showing a statistically significant increasing trend overall (*P* = 0.0153). NDM carriage among C/T nonsusceptible Enterobacterales was very low, ≤1.3% each year from 2016 to 2021; however, it increased to 4.6% in 2022 and 6.5% in 2023, followed by a reduction to 1.8% in 2024, demonstrating a highly significant increasing trend overall (*P* < 0.001). Conversely, the of KPC detection exhibited a highly significant decreasing trend over the 9-year time frame (*P* < 0.001), ranging from a high of 18.1% in 2018 to a low of 6.2% in 2021. Rates of OXA-48-like enzyme detection [range: 0% (2016, 2017 and 2018) to 1.7% (2019)] and acquired AmpC detection [range: 3.7% (2023) to 7.2% (2019)] did not exhibit any significant increasing or decreasing trend over the 9-year time frame.

## Discussion

In this longitudinal analysis of US SMART isolates collected from 2016 through 2024, C/T retained high overall *in vitro* activity against both Enterobacterales and *Pseudomonas aeruginosa*, but temporal patterns differed by pathogen group. The most notable findings were the sustained activity of C/T against *P. aeruginosa*, while a modest but statistically significant decline in C/T susceptibility among Enterobacterales was detected that paralleled increasing detection of CTX-M- and NDM-type β-lactamases among C/T-nonsusceptible isolates. C/T demonstrated consistently potent activity against *P. aeruginosa*, with overall susceptibility of 95.8% and no evidence of declining activity over time when all sites were considered. Indeed, when restricting analysis to consistently participating sites, a modest but statistically significant increase in susceptibility was observed. These findings build upon prior US surveillance reports demonstrating sustained activity of C/T against *P. aeruginosa*, including multidrug-resistant (MDR) and difficult-to-treat resistant (DTR) subsets.^3,16,17^ The preserved activity of C/T likely reflects the stability of ceftolozane against common resistance mechanisms in *P. aeruginosa*, including AmpC overexpression and efflux-mediated resistance, which compromise other β-lactams such as cefepime and piperacillin/tazobactam.^18–22^

In the present study, C/T retained activity against a substantial proportion of resistant phenotypes, inhibiting >80% of isolates nonsusceptible to key antipseudomonal β-lactams and approximately 69% of DTR isolates, underscoring its clinical utility in settings where treatment options are limited. These findings align with contemporary clinical guidance emphasizing C/T as a preferred option for infections due to DTR *P. aeruginosa.*^1^ These data suggest that C/T has remained one of the most reliable β-lactam options for *P. aeruginosa* in the United States, even as susceptibility to older antipseudomonal agents has been lower and more variable.

Recent clinical studies also support the *in vitro* findings observed here. In the multicentre US CACTUS study, ceftolozane/tazobactam was associated with higher clinical success than ceftazidime/avibactam for invasive MDR *P. aeruginosa* infections, with the difference driven largely by pneumonia cases.^23^ In another recent multicentre study, emergence of resistance during therapy was reported less often with ceftolozane/tazobactam than with ceftazidime/avibactam among patients with MDR *P. aeruginosa* bacteremia or pneumonia.^24^ Although clinical outcome studies are not directly comparable to *in vitro* surveillance, they reinforce the practical relevance of the persistently high activity of C/T against US *P. aeruginosa* observed in the present dataset.

In contrast, Enterobacterales showed a different pattern. Overall susceptibility of 93.9% was high, but the significant year-over-year decline, observed both in the full dataset and in the subset of consistently participating sites suggests an epidemiologic shift rather than an artifact of site turnover. The absence of a parallel decline in meropenem susceptibility suggests that the main drivers of reduced C/T activity are not broad increases in carbapenem resistance *per se*, but more likely changes in the prevalence of resistance mechanisms that are less effectively inhibited by tazobactam or that occur in combination with permeability and expression effects. The increasing detection of CTX-M among nonsusceptible isolates is consistent with the continuing central role of CTX-M enzymes in ESBL epidemiology. Although tazobactam can inhibit many CTX-M enzymes, inhibitory activity may still be reduced in the presence of high-level enzyme expression, co-produced β-lactamases, porin alterations or other accompanying resistance determinants.^20,25^ The concomitant rise in NDM detection is particularly concerning because metallo-β-lactamases are not inhibited by tazobactam and therefore directly undermine C/T activity. From a clinical standpoint, the observed decrease in KPC detection together with increased NDM detection suggests a meaningful shift in the carbapenemase landscape among the small but important subset of C/T-nonsusceptible Enterobacterales.

The species-level results support this mechanism-based interpretation. C/T remained highly active against *E. coli* and *K. pneumoniae*, but activity was clearly lower against the *E. cloacae* complex and *C. freundii* complex, taxa associated with inducible chromosomal AmpC production. Tazobactam has limited ability to reliably inhibit AmpC-mediated resistance, which may contribute to the reduced activity observed in these organism groups.^26^

The increase in NDM seen in this study is also concordant with recent US public health reports. CDC investigators reported a sharp rise in NDM-producing CRE in the USA from 2019 to 2023,^27^ and a 2025 MMWR report from New York City documented an increase in annual NDM-positive CRE cases from 58 in 2019 to 388 in 2024, with NDM surpassing KPC in 2024.^28^ While the SMART dataset is not designed to estimate resistance mechanism prevalence in the broader population, the increase in NDM detection among C/T-nonsusceptible Enterobacterales in the present study strongly suggests that it is capturing the same emerging signal as the CDC reports. This trend has important therapeutic implications because increasing MBL incidence reduces the utility of nearly all β-lactam/β-lactamase inhibitor agents and makes accurate detection of underlying carbapenemase class increasingly important for treatment selection.

This study has several strengths, including the large isolate collection, long longitudinal timeframe (nine years), broad multicentre US sampling, centralized broth microdilution testing, and the use of both all-site and consistently contributing-site analyses to address possible bias from changing site participation. The addition of molecular characterization among C/T-nonsusceptible Enterobacterales also provides useful mechanistic context for the observed phenotypic trends. At the same time, several limitations deserve mention. Site participation varied over time, bloodstream isolates were added only beginning in 2018, and not all C/T-nonsusceptible Enterobacterales underwent molecular characterization in every year. Additionally, the statistical methods employed may not capture nuanced variability. The Cochran–Armitage test assumes a single linear trend (essentially asking if there an overall increase or decrease across the years). This disadvantage is partially offset by the Joinpoint method that can identify trend changes over the study period. However, in our case, this analysis identified significant join-points in both Enterobacterales and *P. aeruginosa* C/T susceptibility datasets that included all isolates but not in the sets limited to isolates collected from the consistently contributing sites, raising the question of whether the trend shifts could be an artifact of site participation. Finally, any surveillance dataset based on *in vitro* susceptibility cannot determine clinical efficacy. Nevertheless, the consistency of the major findings with recent surveillance, guidelines and clinical outcome studies supports the validity and relevance of the observed trends.

In summary, C/T maintained excellent activity against US *P. aeruginosa* isolates from 2016 to 2024, including important resistant subsets, supporting its continued role as a key option for challenging pseudomonal infections. By contrast, C/T susceptibility among Enterobacterales declined modestly but significantly over the same period, likely reflecting evolving β-lactamase epidemiology, particularly increasing CTX-M and NDM detection among nonsusceptible isolates. Continued surveillance and mechanism-informed interpretation of susceptibility results will be essential to preserve the clinical utility of C/T and to guide appropriate use as resistance patterns continue to evolve.

## Supplementary Material

dlag134_Supplementary_Data

## Data Availability

Data presented in this manuscript is available from the corresponding author upon reasonable request.

## Transparency declarations

MGW and DFS are employees of IHMA, which receives funding from Merck Sharp & Dohme LLC, a subsidiary of Merck & Co., Inc., Rahway, NJ, USA for the SMART global surveillance program. KB, JE, FS, KY, and MRM are employees of Merck Sharp & Dohme LLC, a subsidiary of Merck & Co., Inc., Rahway, NJ, USA and own stock and options in Merck & Co., Inc., Rahway, NJ, USA. The IHMA authors do not have personal financial interests in the sponsor of this work (Merck Sharp & Dohme LLC, a subsidiary of Merck & Co., Inc., Rahway, NJ, USA).

## Supplementary data

Figures S1 and S2 and Tables S1 to S4 are available as Supplementary data at *JAC-AMR* Online.
