# Supplementary material for: Long–term trends in ceftolozane–tazobactam susceptibility among gram–negative pathogens in the United States: a nine–year SMART analysis (2016–2024)
Source: JAC Antimicrob Resist. 2026 Jul 7;8(4):dlag134. doi: 10.1093/jacamr/dlag134 (PMC13340461; doi:10.1093/jacamr/dlag134)

SUPPLEMENTARY DATA

Table S1. Number of Enterobacterales and *P. aeruginosa* isolates collected for the SMART program by US state and clinical site, 2016-2024.

| State/Site^a^ | 2016 | 2017 | 2018 | 2019 | 2020 | 2021 | 2022 | 2023 | 2024 | Grand Total |
| --- | --- | --- | --- | --- | --- | --- | --- | --- | --- | --- |
| **AL** |  |  |  |  |  |  |  |  |  |  |
| Site A |  |  |  |  |  |  | 134 | 58 |  | **192** |
| **AZ** |  |  |  |  |  |  |  |  |  |  |
| Site A |  |  |  |  |  | 214 | 187 | 208 |  | **609** |
| **CA** |  |  |  |  |  |  |  |  |  |  |
| Site A | 238 | 235 | 164 | 218 | 226 | 200 | 178 | 191 | 200 | **1850** |
| Site B | 243 | 222 | 175 | 218 | 204 | 193 | 182 | 169 | 281 | **1887** |
| **CO** |  |  |  |  |  |  |  |  |  |  |
| Site A | 233 | 233 | 220 | 232 | 238 | 240 | 234 | 231 | 235 | **2096** |
| **FL** |  |  |  |  |  |  |  |  |  |  |
| Site A |  |  |  |  |  |  | 232 | 231 | 216 | **679** |
| Site B | 234 | 182 | 97 | 239 | 180 | 228 | 62 |  | 212 | **1434** |
| Site C | 203 | 226 | 110 |  |  |  |  |  |  | **539** |
| **GA** |  |  |  |  |  |  |  |  |  |  |
| Site A |  |  | 194 | 233 | 228 | 233 | 138 |  |  | **1026** |
| Site B | 181 | 146 |  |  |  |  |  |  |  | **327** |
| **IA** |  |  |  |  |  |  |  |  |  |  |
| Site A | 97 |  |  |  |  |  |  |  |  | **97** |
| **IL** |  |  |  |  |  |  |  |  |  |  |
| Site A | 221 | 230 | 231 | 225 | 225 | 229 | 222 | 217 | 220 | **2020** |
| Site B |  |  | 241 | 233 | 235 | 230 | 197 | 189 | 219 | **1544** |
| **IN** |  |  |  |  |  |  |  |  |  |  |
| Site A | 233 | 245 | 161 | 231 | 233 |  |  |  |  | **1103** |
| Site B | 246 | 247 | 252 | 239 | 192 | 232 | 217 |  |  | **1625** |
| **KY** |  |  |  |  |  |  |  |  |  |  |
| Site A | 237 | 225 |  |  |  |  |  |  | 210 | **672** |
| **MI** |  |  |  |  |  |  |  |  |  |  |
| Site A | 226 | 230 | 232 | 234 | 233 | 239 |  | 203 | 227 | **1824** |
| **MN** |  |  |  |  |  |  |  |  |  |  |
| Site A |  | 223 | 232 |  |  |  |  |  |  | **455** |
| **NC** |  |  |  |  |  |  |  |  |  |  |
| Site A | 235 | 195 | 189 | 222 | 198 |  |  |  |  | **1039** |
| **ND** |  |  |  |  |  |  |  |  |  |  |
| Site A |  |  |  |  |  |  |  |  | 95 | **95** |
| **NE** |  |  |  |  |  |  |  |  |  |  |
| Site A |  | 160 | 123 | 218 |  |  |  |  |  | **501** |
| **NM** |  |  |  |  |  |  |  |  |  |  |
| Site A |  |  |  |  |  |  |  | 121 | 172 | **293** |
| **NY** |  |  |  |  |  |  |  |  |  |  |
| Site A |  |  |  |  |  |  |  |  | 226 | **226** |
| Site B | 232 | 233 | 111 | 204 | 224 |  |  |  |  | **1004** |
| Site C | 232 | 213 | 243 | 242 | 233 | 243 | 223 | 209 | 201 | **2039** |
| Site D | 141 | 226 | 233 | 219 | 218 | 170 | 103 | 101 | 138 | **1549** |
| **OH** |  |  |  |  |  |  |  |  |  |  |
| Site A | 226 | 232 | 220 |  |  |  |  |  |  | **678** |
| Site B | 181 | 173 | 209 | 211 | 212 | 223 | 215 | 217 | 228 | **1869** |
| **PA** |  |  |  |  |  |  |  |  |  |  |
| Site A | 35 | 86 |  |  |  |  |  |  |  | **121** |
| **TN** |  |  |  |  |  |  |  |  |  |  |
| Site A |  |  |  |  |  | 230 | 98 | 223 | 108 | **659** |
| **TX** |  |  |  |  |  |  |  |  |  |  |
| Site A |  |  | 236 | 238 | 242 | 254 |  |  |  | **970** |
| Site B |  |  |  |  |  | 193 | 155 | 218 | 227 | **793** |
| **UT** |  |  |  |  |  |  |  |  |  |  |
| Site A |  | 206 | 220 | 239 | 231 | 234 | 230 | 229 | 257 | **1846** |
| **VA** |  |  |  |  |  |  |  |  |  |  |
| Site A |  |  |  |  |  |  | 202 | 130 |  | **332** |
| **WA** |  |  |  |  |  |  |  |  |  |  |
| Site A | 233 | 203 | 241 | 214 | 224 | 231 | 223 | 209 | 215 | **1993** |
| **WI** |  |  |  |  |  |  |  |  |  |  |
| Site A |  | 109 | 82 | 51 | 26 |  |  |  |  | **268** |
| Site B | 209 | 209 | 137 | 224 | 223 | 211 | 208 | 219 | 218 | **1858** |
| **Grand Total** | **4316** | **4889** | **4553** | **4584** | **4225** | **4227** | **3640** | **3573** | **4105** | **38112** |

^a^ Site information anonymized with letter designation.

Table S2. Longitudinal trends from 2016 to 2024 in the percentage of Enterobacterales isolates testing as susceptible to the indicated agents among (A) all isolates collected in the U.S. for the SMART program and (B) isolates collected from solely the clinical sites that participated in the program each year from 2016 to 2024.. Data correspond to Figure 1 in main body of manuscript.

A)

| Year (n) | % susceptible | | | |
| --- | --- | --- | --- | --- |
|  | Ceftolozane/tazobactam | Meropenem | Cefepime | Piperacillin/tazo-bactam |
| 2016 (3420) | 93.9 | 98.7 | 89.2 | 86.6 |
| 2017 (3898) | 94.1 | 98.5 | 88.7 | 86.3 |
| 2018 (3657) | 95.3 | 98.9 | 90.8 | 87.4 |
| 2019 (3717) | 95.1 | 99.1 | 90.6 | 89.8 |
| 2020 (3408) | 94.1 | 99.2 | 88.2 | 87.3 |
| 2021 (3386) | 93.3 | 98.8 | 88 | 86.1 |
| 2022 (2956) | 94.1 | 99.1 | 88 | 87.5 |
| 2023 (2967) | 92.2 | 98.3 | 84.6 | 86.8 |
| 2024 (3249) | 92 | 98.4 | 83.7 | 85.6 |

B)

| Year (n) | % susceptible | | | |
| --- | --- | --- | --- | --- |
|  | Ceftolozane/tazobactam | Meropenem | Cefepime | Piperacillin/tazo-bactam |
| 2016 (1537) | 93.1 | 98.4 | 89.4 | 85.4 |
| 2017 (1573) | 94 | 98.3 | 88.9 | 86.2 |
| 2018 (1480) | 93.2 | 98.4 | 89.9 | 84.9 |
| 2019 (1609) | 94.5 | 99.1 | 89.3 | 89.3 |
| 2020 (1608) | 92.2 | 98.9 | 87.6 | 85 |
| 2021 (1559) | 92.8 | 99 | 88.8 | 85.6 |
| 2022 (1464) | 93.8 | 98.9 | 89.3 | 87.6 |
| 2023 (1457) | 91.9 | 98.8 | 88.3 | 87.2 |
| 2024 (1544) | 91.4 | 98.5 | 87.4 | 85.9 |

Table S3. Longitudinal trends from 2016 to 2024 in the percentage of *P. aeruginosa* isolates testing as susceptible to the indicated agents among (A) all isolates collected in the U.S. for the SMART program and (B) isolates collected from solely the clinical sites that participated in the program each year from 2016 to 2024. Data correspond to Figure 2 in main body of manuscript.

A)

| Year (n) | % susceptible | | | |
| --- | --- | --- | --- | --- |
|  | Ceftolozane/tazobactam | Meropenem | Cefepime | Piperacillin/tazo-bactam |
| 2016 (896) | 95 | 78.2 | 73.8 | 71.2 |
| 2017 (991) | 94.5 | 75.8 | 77.8 | 69.3 |
| 2018 (896) | 96.4 | 76.9 | 78.8 | 73.2 |
| 2019 (867) | 96.2 | 78 | 83.5 | 79.5 |
| 2020 (817) | 96.9 | 79.9 | 82.7 | 78.7 |
| 2021 (841) | 96.8 | 80.4 | 80.7 | 77.5 |
| 2022 (684) | 96.3 | 79.5 | 81.7 | 75.4 |
| 2023 (610) | 95.1 | 77.9 | 78.7 | 74.3 |
| 2024 (856) | 95 | 77.5 | 79.7 | 76.2 |

B)

| Year (n) | % susceptible | | | |
| --- | --- | --- | --- | --- |
|  | Ceftolozane/tazobactam | Meropenem | Cefepime | Piperacillin/tazo-bactam |
| 2016 (394) | 93.1 | 77.9 | 72.1 | 70.1 |
| 2017 (371) | 91.9 | 71.7 | 72.5 | 65.8 |
| 2018 (373) | 95.4 | 74.3 | 75.1 | 71.3 |
| 2019 (394) | 94.4 | 78.4 | 83.2 | 80.5 |
| 2020 (395) | 95.2 | 74.9 | 80 | 77.7 |
| 2021 (381) | 95.8 | 77.7 | 77.4 | 74.3 |
| 2022 (324) | 96 | 74.4 | 82.7 | 73.8 |
| 2023 (306) | 93.8 | 81.4 | 79.7 | 75.5 |
| 2024 (391) | 96.4 | 79.5 | 81.1 | 79.3 |

Table S4. Acquired β-lactamase detection rate among characterized C/T-non-susceptible Enterobacterales in the U.S., 2016-2024. Data correspond to Figure 3 in main body of manuscript.

| Year (n) | Detection rate | | | | |
| --- | --- | --- | --- | --- | --- |
|  | NDM | KPC | OXA-48-like | CTX-M | Acquired AmpC |
| 2016 (207) | 0.0% | 15.0% | 0.0% | 16.4% | 6.8% |
| 2017 (228) | 0.4% | 15.4% | 0.0% | 13.2% | 6.6% |
| 2018 (160) | 1.3% | 18.1% | 0.0% | 15.6% | 5.6% |
| 2019 (181) | 0.0% | 13.8% | 1.7% | 14.9% | 7.2% |
| 2020 (182) | 0.5% | 10.4% | 1.6% | 14.8% | 4.9% |
| 2021 (210) | 1.0% | 6.2% | 0.5% | 13.8% | 4.3% |
| 2022 (174) | 4.6% | 9.2% | 1.1% | 14.4% | 5.2% |
| 2023 (216) | 6.5% | 9.3% | 0.9% | 27.8% | 3.7% |
| 2024 (218) | 1.8% | 10.6% | 0.5% | 17.9% | 5.0% |

Figure S1. Joinpoint regression program output for nine-year ceftolozane/tazobactam annual percentage susceptibility trends among clinical isolates of Enterobacterales from all clinical sites (A), and solely the sites participating each year (B).

(A)


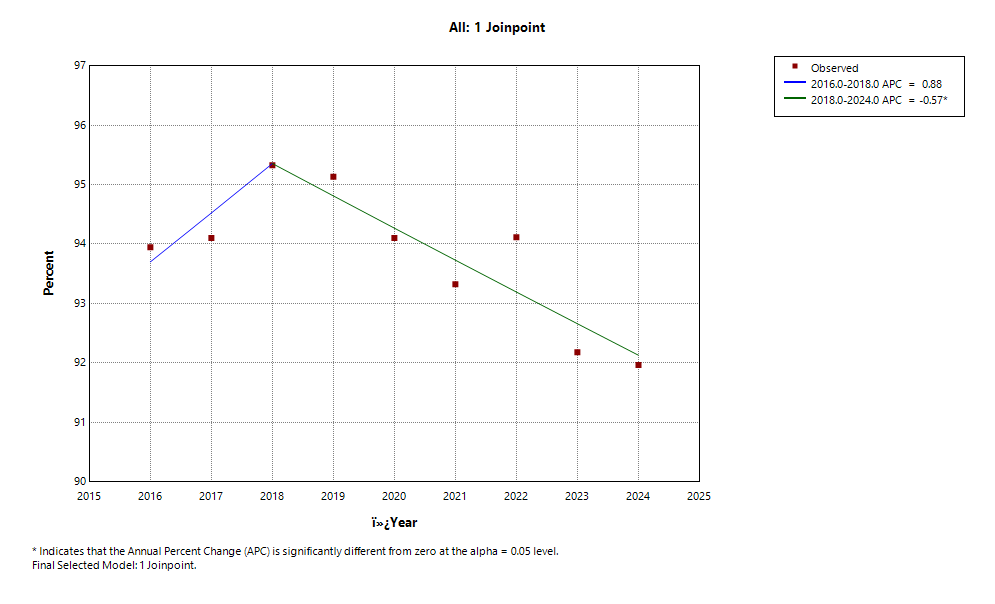


(B)


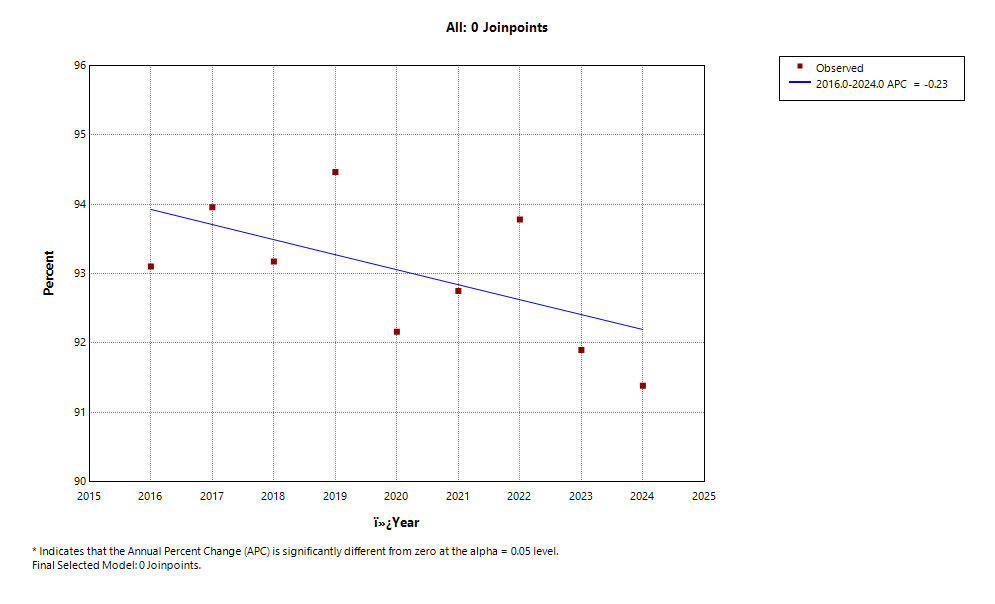


Figure S2. Joinpoint regression program output for nine-year ceftolozane/tazobactam annual percentage susceptibility trends among clinical isolates of *P. aeruginosa* from all clinical sites (A), and solely the sites participating each year (B).

(A)


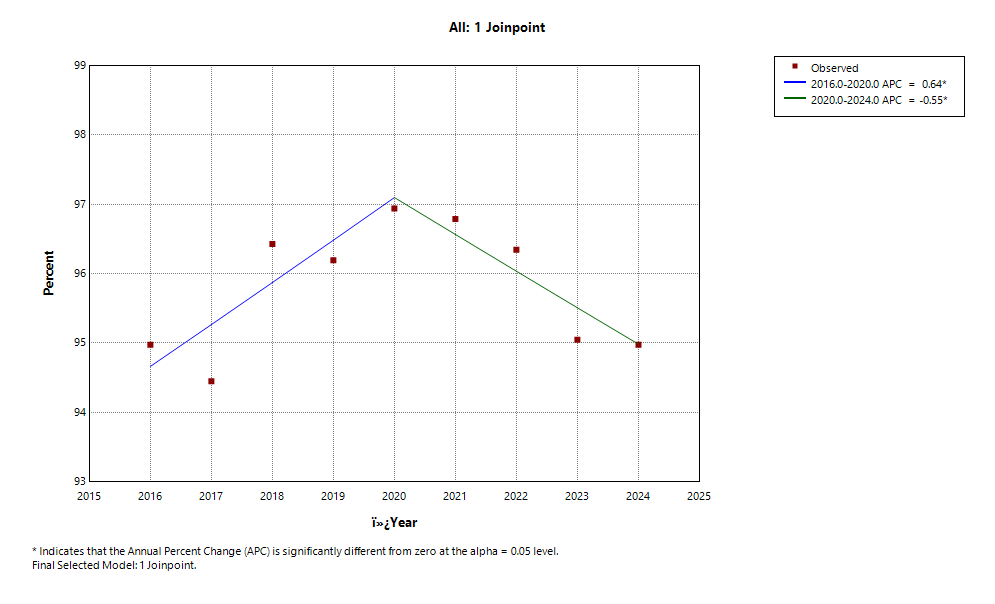


(B)


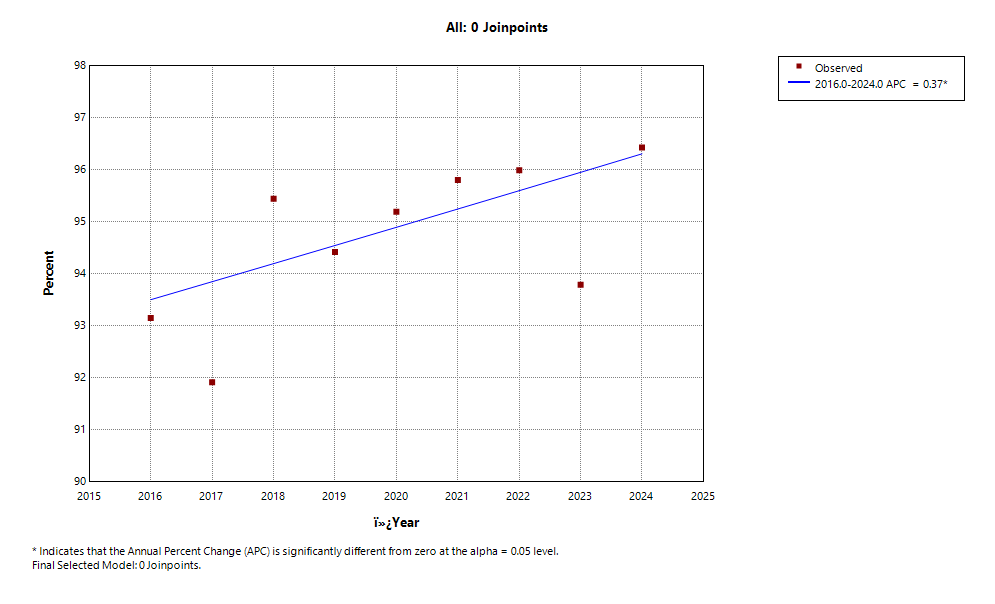

Supplement: dlag134_Supplementary_Data [file dlag134_supplementary_data.docx]
